# Characterization of a stable HIV-1 B/C recombinant, soluble, and trimeric envelope glycoprotein (Env) highly resistant to CD4-induced conformational changes

**DOI:** 10.1074/jbc.M117.803056

**Published:** 2017-07-25

**Authors:** Rajesh Kumar, Gabriel Ozorowski, Vivek Kumar, Lauren G. Holden, Tripti Shrivastava, Shilpa Patil, Suprit Deshpande, Andrew B. Ward, Jayanta Bhattacharya

**Affiliations:** From the ‡ HIV Vaccine Translational Research Laboratory, Translational Health Science and Technology Institute, National Capital Region Biotech Science Cluster, Faridabad, Haryana 121001, India,; § Department of Integrative Structural and Computational Biology, Center for HIV/AIDS Vaccine Immunology and Immunogen Discovery, International AIDS Vaccine Initiative Neutralizing Antibody Center and Collaboration for AIDS Vaccine Discovery, The Scripps Research Institute, La Jolla, California 92037, and; ¶ International AIDS Vaccine Initiative, New York, New York 10004

**Keywords:** cluster of differentiation 4 (CD4), human immunodeficiency virus (HIV), viral protein, viral replication, virus entry, B/C recombinant, SOSIP.664, broadly neutralizing antibodies, envelope, trimeric protein

## Abstract

The HIV-1 envelope (Env) is a glycoprotein consisting of a trimer of heterodimers containing gp120 and gp41 subunits that mediates virus entry and is a major target of broadly neutralizing antibodies (bnAbs) developed during infection in some individuals. The engagement of the HIV-1 gp120 glycoprotein to the host CD4 protein triggers conformational changes in gp120 that allow its binding to co-receptors and is necessary for virus entry to establish infection. Native-like HIV-1 Env immunogens representing distinct clades have been proposed to improve immunogenicity. In the present study, we examined the basis of resistance of an HIV-1 B/C recombinant Env (LT5.J4b12C) to non-neutralizing antibodies targeting CD4-induced Env epitopes in the presence of soluble CD4 (sCD4). Using native polyacrylamide gel shift assay and negative-stain EM, we found that the prefusion conformational state of LT5.J4b12C trimeric Env was largely unaffected in the presence of excess sCD4 with most Env trimers appearing to be in a ligand-free state. This resistance to CD4-induced conformational changes was associated with a lower affinity for CD4. Moreover, the LT5.J4b12C trimeric Env preferentially bound to the neutralizing antibodies compared with non-neutralizing antibodies. Taken together, we report on an HIV-1 B/C recombinant, native-like trimeric Env protein that is highly resistant to CD4-induced conformational changes but displays epitopes recognized by a diverse array of bnAbs. Such features make this B/C recombinant trimeric Env a useful addition to the pool of other recently identified native-like HIV-1 Env trimers suitable for use as antigenic bait for bnAb isolation, structural studies, and use as potential immunogens.

## Introduction

The human immunodeficiency virus, type 1 (HIV-1)^2^ envelope (Env) glycoprotein mediates virus entry and is a major target of broadly neutralizing antibodies (bnAbs) developed during the course of infection in a subset of individuals (1). Env has a high degree of intrinsic flexibility and undergoes a major conformational change upon engagement with its primary receptor, CD4 on helper T cells, resulting in exposure of the co-receptor (CXCR4/CCR5)-binding site (CoRbs) necessary for receptor-mediated endocytosis leading to viral entry. Although the *modus operandi* of the HIV-1 entry mediated by Env is defined, the inter- and intraclade sequence diversities, difficulties in stable presentation of well-ordered native gp120–gp41 subunits in soluble form, and immunization strategies are collectively believed to be impeding the progress of developing a successful immunogen capable of eliciting bnAbs (2–6). Recent progress in stabilization of codon-optimized trimeric Env proteins by selective modification of Env (gp140) sequences (7–11) has provided an opportunity to better understand the structural and antigenic properties of well-ordered soluble Env proteins in their native state. One design, SOSIP Env, which mimics native viral trimers, has been shown to preferentially recognize bnAbs by virtue of presenting their epitopes while reducing those that are targeted by non-neutralizing antibodies (12, 13). Nonetheless, recent studies have shown that SOSIP trimers vary in flexibility and conformational states depending on the genotype of the parent virus (14, 15). For example, the clade A BG505 SOSIP.664 Env was found to adopt a more compact conformation compared with other SOSIP counterparts such as B41 (clade B) and DU422 and ZM197M (clade C) (16, 17). The degree of stabilization minimizing the Env flexibility that modulates its conformation during physiologically relevant events such as CD4 engagement is important to prevent exposure of immunodominant epitopes that are targets of non-neutralizing antibodies. This is particularly important to impede the generation of non-neutralizing antibodies that may hinder engagement of B cells having specificity for bnAb epitopes (18, 19). Improved strategies to stabilize HIV-1 Env trimers have been reported (8, 20, 21), and those with reduced CD4 affinity have recently been demonstrated to show improved immunogenicity in animal models (7, 22).

Variations in immune response to HIV-1 have been reported within and between populations that govern the selection of viral variants and escape mutants via selective substitutions of amino acid residues, modification of glycosylation patterns in Env, and recombination events (23). Viral surface glycoproteins (such as Env), which may be desirable for vaccine regimens, are themselves very diverse. The diversity of viral variants circulating in ethnically distinct host populations may impede the elicitation of the desired protective immune response in populations in whom the vaccine would be tested (24). Hence, in addition to engineering Env for its structural stability in equilibrium state, a deep understanding of variation of humoral immune response in different infected populations will help select Env backbones for rational design of immunogens that may elicit bnAb responses in a targeted population.

We previously reported (25) an HIV-1 B/C recombinant chimeric Env (LT5.J4b12C) prepared using two autologous *envs* obtained from broadly cross-neutralizing plasma of an antiretroviral therapy-naïve slow progressing Indian patient (26) that when expressed as pseudotyped virus showed resistance to a number of non-neutralizing mAbs, including those that target the CoRbs. Interestingly, although LT5.J4b12C Env was found to be sensitive to CD4bs mAb b12, it showed resistance to VRC01 and sCD4 (25). In the present study, we characterized the antigenic properties of the soluble form of the LT5.J4b12C trimeric Env and investigated the biochemical and structural basis of resistance of this highly stable trimeric Env to CD4-induced conformational changes associated with its inability to form stable complexes with CD4.

## Results

### A B/C recombinant HIV-1 Env is refractory to CD4-induced exposure of neutralizing epitopes when expressed as pseudotyped virus

We previously reported association of unique sequences in the C2V3 region of the HIV-1 B/C recombinant primary Env obtained from cross-neutralizing plasma with increased sensitivity to autologous broadly cross-neutralizing plasma antibodies (25). In addition, we also reported that the LT5.J4b (expressing C2V3 sequence of the contemporaneous LT5.J12 Env or LT5.J4b12C) to be susceptible to autologous plasma antibodies b12 and 4E10 mAbs; however, it was found to be resistant to sCD4, VRC01, 17b, and 3074. Because LT5.J4b12C Env was found earlier to be resistant to both sCD4 and 17b mAb, in the present study, we further examined whether sCD4 could confer susceptibility of this Env to CD4i epitope-directed antibodies. Pseudovirus expressing LT5.J4b12C Env pretreated with 25 μg/ml sCD4 was resistant to CD4i epitope-directed mAbs such as 17b, X5, 412D, and A324 (Table 1). YU2 was used as a positive control (data not shown). Our data substantiate our previous observation and further indicated that, due to its intrinsic resistance to sCD4, the LT5.J4b12C Env was unable to undergo conformational changes required for exposure of CD4i epitopes. Interestingly, pseudoviruses expressing LT5.J4b12C Env were found to be neutralized by a number of bnAbs with distinct specificities such as b12, VRC03, PG9, PGT121, and PGT151 (Table 2). This observation along with the resistance to non-neutralizing antibodies such as 17b and 3074 noted in the present and previous (25) studies made LT5.J4b12C an attractive Env to explore further and probe whether the properties are retained when expressed as a soluble trimeric protein.

**Table 1:** **Effect of sCD4 on neutralization of pseudotyped virus expressing LT5.J4b12C Env by CD4i-directed mAbs** Env-pseudotyped virus was pretreated with different doses of sCD4 up to 25 μg/ml at 37 °C for an hour before incubating with different mAbs at 10 μg/ml. Virus neutralization was assessed in duplicates in TZM-bl cells and repeated three times.

Env	Clade	mAb	IC_50_	
			−sCD4	+sCD4
			μ*g*/*ml*	
LT5.J4b12C	B/C	None	None	>25
LT5.J4b12C	B/C	X5	>25	>25
LT5.J4b12C	B/C	412D	>25	>25
LT5.J4b12C	B/C	17b	>25	>25
LT5.J4b12C	B/C	A324	>25	>25

**Table 2:** **Sensitivity of Env-pseudotyped viruses to bnAbs** Values indicate concentration of IgG (μg/ml) conferring 50% virus neutralization in TZM-bl cells. The neutralization assay was done in duplicates and repeated at least three times.

mAbs/entry inhibitor	Target	LT5.J4b12C
b12	CD4bs	1.55
VRC01	CD4bs	>10
VRC03	CD4bs	<0.04
PG9	V2 apex	<0.04
PGT121	V3 base	<0.04
PGT151	gp120-gp41 interface	<0.04
17b	CoRbs	>25
3074	V3 tip	>25
sCD4	CD4bs	>25

### Highly stable and well-ordered native-like soluble LT5.J4b12C Env trimers are highly resistant to CD4-induced conformational changes

**Figure 1.**
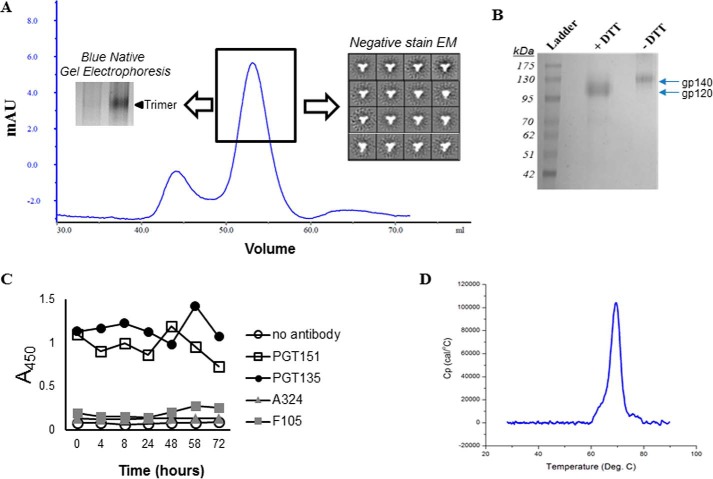
**Purification and characterization of LT5.J4b12C SOSIP.664.** LT5.J4b12C SOSIP.664 trimeric Env was first purified by PGT151 mAb affinity chromatography followed by size exclusion chromatography. *A*, quality of the size exclusion chromatography-purified SOSIP trimer was assessed by negative-stain EM, and expression was assessed by 4–15% blue native gel electrophoresis. *B*, LT5.J4b12C was found to be efficiently cleaved when examined by SDS-PAGE under reducing conditions. *C*, the stability of LT5.J4b12C SOSIP.664 at 37 °C as a function of time was examined by incubating the trimers for 72 h using a thermal cycler followed by D7324-capture ELISA to assess the extent of binding of the trimers collected at different time points to the bnAbs (PGT135 and PGT151) and non-binding mAbs (F105, 48D, and A324). *D*, differential scanning calorimetry analysis of LT5.J4b12C SOSIP.664. *mAU*, milliabsorbance units; *deg*, degrees; *Cp*, specific heat capacity.

To assess the effect of CD4 presence on the degree of conformational changes in the LT5.J4b12C trimeric protein, we first prepared codon-optimized soluble LT5.J4b12C SOSIP.664 Env protein by incorporating previously described modifications (11) and characterized its biophysical properties. The LT5.J4b12C SOSIP.664 was purified using a PGT151 affinity column followed by size exclusion chromatography to obtain homogenous, well-ordered trimers (Fig. 1*A*) as observed by negative-stain EM analysis. Efficient furin cleavage was confirmed by reducing SDS-polyacrylamide gel electrophoresis (PAGE) (Fig. 1*B*), and LT5.J4b12C SOSIP.664 was found to remain stable at 37 °C for at least 72 h (Fig. 1*C*). The thermal stability of LT5.J4b12C SOSIP.664 was also measured using differential scanning calorimetry (DSC). The midpoint of thermal denaturation (*T_m_*) for LT5.J4b12C SOSIP.664 trimer was found to be 69.4 °C (Fig. 1*D*), indicating that it is highly stable.

**Figure 2.**
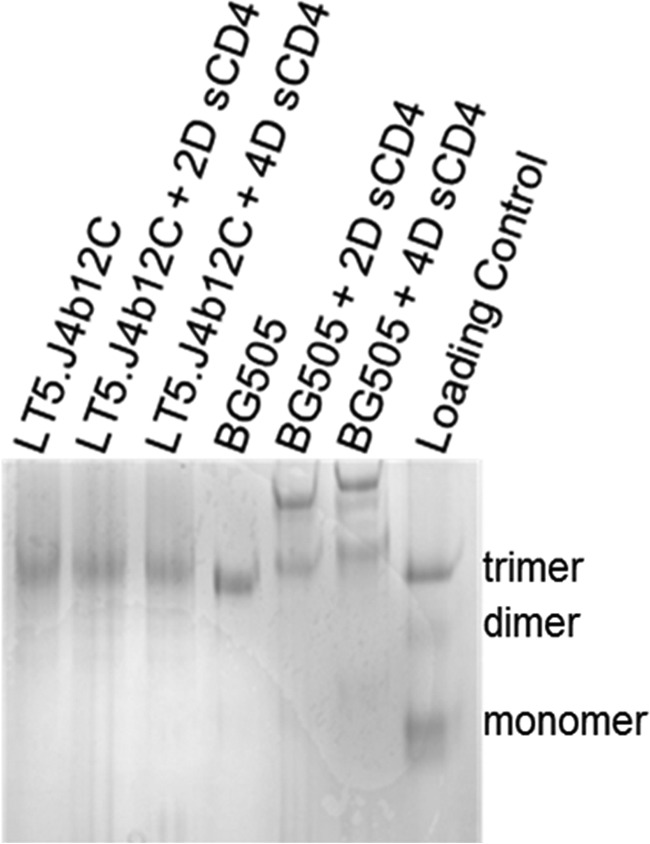
**BN-PAGE gel shift assay.** BN-PAGE analysis of SOSIPs of LT5.J4b12C and BG505 (2.5 μm each) was done in the presence and absence of a 10-fold molar excess concentration of sCD4 (25 μm). *2D* and *4D sCD4* denotes two- and four-domain sCD4, respectively.

To examine any conformational differences between ligand-free and CD4-bound LT5.J4b12C SOSIP.664, we carried out blue native (BN) PAGE gel shift assays on unliganded LT5.J4b12C SOSIP.664 and LT5.J4b12C SOSIP.664 preincubated with an excess amount of sCD4 (both two-domain and four-domain sCD4). BG505 SOSIP.664 (both unliganded and in CD4-bound state) was also run in parallel to serve as an assay control because it was previously shown to bind to CD4 in a saturated manner (7). In sharp contrast to BG505 SOSIP.664, we found neither dimerization of the trimer nor higher order oligomer formation of the LT5.J4b12C SOSIP.664 Env (Fig. 2). Overall, our data indicate that the trimeric soluble Env largely retains its prefusion conformation in the presence of exogenously added sCD4.

### Reduced exposure of CD4-induced epitopes in LT5.J4b12C trimeric Env is associated with intrinsic resistance to CD4-induced conformational change

**Figure 3.**
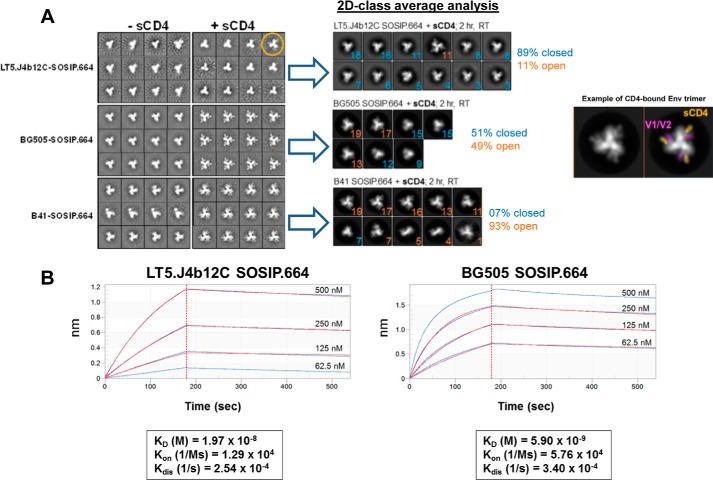
**Biophysical characterization of LT5.J4b12C SOSIP.664.** *A*, two-dimensional negative-stain EM class averages of various SOSIP.664 trimers, ligand-free or in complex with a 6-fold molar excess of sCD4 (2-h incubation at room temperature at concentrations of 1.4 μm for SOSIP and 9 μm for sCD4). Percentages of particles belonging to a specific class (open and closed conformations) are shown. An example of CD4-bound class with false coloring (*bottom*) is shown or reference. *B*, biolayer interferometry kinetics analysis of SOSIP trimer binding to sCD4. Fitted curves (1:1 binding model) are shown in *red*.

To substantiate this observation, we examined the conformational states of LT5.J4b12C SOSIP.664 in the absence and presence of excess sCD4 by two-dimensional negative-stain electron microscopy (EM) analysis. BG505 SOSIP.664 and B41 SOSIP.664 were assessed in parallel under the same experimental conditions. Although ligand-free LT5.J4b12C SOSIP.664 forms compact and closed trimers comparable with those of B41 SOSIP.664, a significant difference in conformational shift was observed between these two SOSIP Envs postincubation with a 6-fold molar excess of sCD4 at room temperature for 2 h (Fig. 3*A*). Two-dimensional class average analysis of trimer populations revealed that a majority of the (∼90%) (Fig. 3*A*) of LT5.J4b12C SOSIP.664 trimers remained in closed prefusion state post-treatment with sCD4. Overall, our data suggest that LT5.J4b12C trimeric Env is highly resistant to CD4-induced conformational change.

To establish whether this increased resistance of LT5.J4b12C SOSIP.664 trimers to undergo CD4-induced conformational changes is due to low affinity for CD4, we next assessed the kinetics of the binding of LT5.J4b12C SOSIP.664 to CD4. Dose-dependent binding of SOSIP trimers to His-tagged sCD4 immobilized to Ni-NTA sensors were analyzed by biolayer interferometry (BLI) Octet analysis. LT5.J4b12C SOSIP.664 was found to bind to sCD4 with ∼3-fold lower affinity (1.97 × 10^−8^) with a slower on-rate and faster off-rate compared with BG505 SOSIP.664 under the same experimental conditions (Fig. 3*B*). Our data indicate that increased resistance of LT5.J4b12C SOSIP.664 to CD4-induced conformational changes is associated with less favorable binding kinetics to CD4.

### The prefusion LT5.J4b12C Env conformation is associated with loss of CD4 and VRC01 recognition

**Figure 4.**
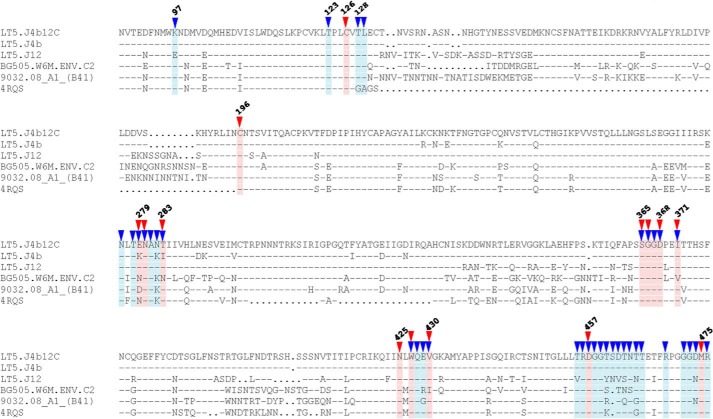
**Amino acid sequence alignment of LT5.J4b12C with BG505, B41, LT5.J4b, LT5.J12, and YU2 (4RQS) Envs.** The CD4 and VRC01 mAb contact points are marked by *red* and *blue arrows.* The amino acid residue numbering was done based on HxBc2 sequence. Residues 371, 430, and 475 are involved in hydrophobic interactions; residues 126, 196, 279, 280, 283, 365, 366, and 425 form main chain–main chain, main chain–side chain, and side chain–side chain hydrogen bonds. Asp^457^ is involved in an ionic interaction with CD4.

**Figure 5.**
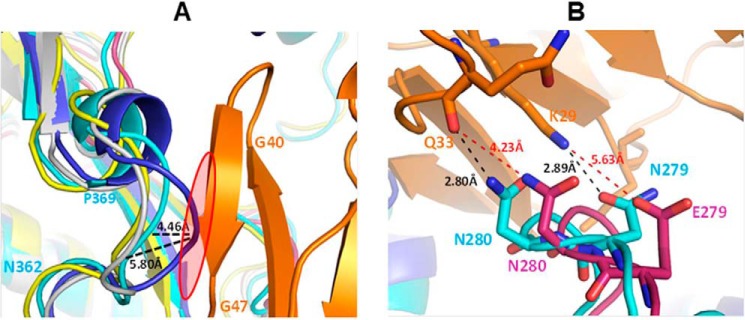
**Structure-based prediction for resistance of LT5.J4b12C toward CD4 binding.** *A*, predicated conformations of neutralization-sensitive LT5.J4b (*white*) and chimeric LT5.J4b12C (*blue*) Env structures were superimposed with the CD4 (*orange*)-bound conformation of YU2 (Protein Data Bank code 4QRS; *cyan*) and BG505 (Protein Data Bank code 5THR). The spatial orientation of loop 362–369 of LT5.J4b12C shows displacement toward CD4 compared with Protein Data Bank code 4QRS and LT5.J4b. The marked highlighted *red circle* shows probable steric clash and possible hindrance in CD4 binding. *B*, analysis of CD4 contact points (Protein Data Bank code 4QRS) with the C2V3 (*pink*) region of the LT5.J4b12C Env (*blue*). The structural orientation of loop D indicates possible changes in interactions between LT5.J4b12C Env and CD4 (Asn^280^–Gln^33^ and Glu^279^–Lys^29^).

**Figure 6.**
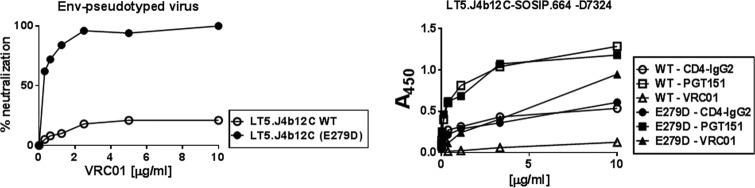
**Effect of E279D on VRC01 and CD4-Ig binding.** *A*, neutralization of pseudotyped virus expressing LT5.J4b12C wild-type (*WT*) and E279D mutant Envs by VRC01 mAb in TZM-bl cells. *B*, binding of LT5.J4b12C SOSIP.664-D7324 to VRC01 and CD4-Ig was measured by D7324-capture ELISA. PGT151 mAb was used to normalize equal amounts of the trimeric form of LT5.J4b12C Env variants used in this assay. For both virus neutralization and SOSIP binding by ELISA, values indicate the mean of two independent experiments done in duplicates.

We next examined the possible mechanisms that are likely associated with unstable interaction of LT5.J4b12C with CD4. Because LT5.J4b12C Env was found to be resistant to both sCD4 (25) and VRC01 mAb when expressed as a pseudovirus and did not bind to VRC01 mAb when expressed as a SOSIP protein, we compared its amino acid sequence with those of BG505 and B41 Envs to examine any differences in residues that form contact points with CD4 and VRC01 mAb (Fig. 5). We observed subtle differences in residues that bind to CD4 and are recognized by VRC01 mAb. For example, LT5.J4b12C Env contains a polar asparagine residue compared with a charged lysine residue present in BG505 and B41 Envs at the 282 position in loop D that is needed for VRC01 recognition. Compared with BG505 and B41, the LT5.J4b12C Env also was found to differ in possessing distinct VRC01-targeting amino acid residues at positions 279 (also CD4bs), 429, 460, and 462 (Fig. 4). Further examination of a homology model prepared by superimposition of LT5.J4b12C Env along with the wild-type LT5.J4b and LT5.J12 Envs (used to prepare the chimeric Env) onto the backbone of a CD4-bound YU2 crystal structure (Protein Data Bank code 4RQS) and BG505 (Protein Data Bank code 5THR) revealed an overall change in conformation of LT5.J4b12C Env (Fig. 5*A*) in the spatial orientation of loop 362–369 in the C3 region of LT5.J4b12C. Because the LT5.J4b12C chimera was prepared by swapping the C2V3 region of LT5.J12 into LT5.J4b Env (25), we predict that this induces a change in Env conformation and results in resistance of LT5.J4b12C Env to VRC01 and sCD4. LT5.J4b12C contains two proline residues in this region (Pro^363^ and Pro^369^) and lacks a glycan at position 362 or 363. The differences at the three amino acid positions make it unique among the compared structures and indicated that the predicted conformational change is plausible. Further amino acid sequence differences in loop D of LT5.J4b12C (Fig. 5*B*), unique to the C2V3 sequence, may be associated with alterations of the gp120–CD4 interface with a potential formation of a salt bridge between Glu^279^ (gp120) and Lys^29^ (CD4) but displacement of a hydrogen bond between the side chain of Asn^280^ (gp120) and backbone carbonyl of Gln^33^ (CD4). Interestingly, with a single E279D substitution, LT5.J4b12C Env was found to become susceptible to VRC01 mAb (Fig. 6*A*); however, it was not found to modulate binding of LT5.J4b12C SOSIP.664 to CD4-Ig (Fig. 6*B*), indicating that natural resistance of LT5.J4b12C Env to VRC01 did not impact its binding to CD4. Other amino acid residues such as Thr^460^ and Asp^462^, however, were found to have no impact on VRC01 recognition or CD4-Ig binding (data not shown), indicating that overall structural alterations and loop displacement (including loop D) possibly result in structural hindrance, which allows for CD4 binding to gp120 but limits the stable and longer lasting engagement of the two molecules.

### LT5.J4b12C SOSIP.664 Env preferentially binds to broadly neutralizing antibodies

**Figure 7.**
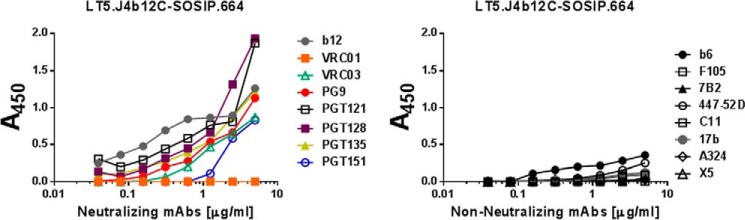
**Antigenic properties of LT5.J4b12C Env protein.** The extent of binding of LT5.J4b12C to neutralizing and non-neutralizing mAbs with distinct specificities was examined by D7324-capture ELISA. Data represent the average of three independent experiments carried out in duplicates.

Finally, we examined the antigenic property of LT5.J4b12C SOSIP.664 by assessing the extent of its binding to both neutralizing and non-neutralizing antibodies by D7324-capture ELISA as described under “Experimental procedures.” We found that LT5.J4b12C SOSIP.664 Env preferentially binds to most of the bnAbs tested than to the non-neutralizing mAbs tested under the same experimental condition (Fig. 7). Our data indicate that the native-like and efficiently cleaved LT5.J4b12C SOSIP.664 Env that forms native trimers presents epitopes that are selectively targeted by bnAbs with distinct specificities. Such properties make LT5.J4b12C SOSIP.664 an attractive antigen that may be used as bait to sort memory B cells isolated from bnAb-producing elite neutralizers.

## Discussion

We previously reported a chimeric HIV-1 B/C recombinant Env (LT5.J4b12C) that when expressed as a pseudovirus was found to be resistant to sCD4 (25). In the present study, we examined the basis of resistance to CD4 of this Env when expressed as a soluble trimeric protein. We show that the inability to form stable complexes with CD4 associated with resistance to CD4-induced conformational changes is an intrinsic property of this highly stable soluble trimeric Env and is not governed by mutations reported recently (7, 22). We previously reported an association between the presence of a unique sequence in the C2V3 region with resistance of a B/C recombinant Env (LT5.J12) isolated from an elite neutralizer to mAbs targeting V3 and CD4bs (VRC01) as well as to sCD4 when expressed as pseudotyped virus (25). This neutralization phenotype was noted by making chimeric Envs between LT5.J12 and a neutralization-sensitive Env (LT5.J4b) obtained from the same individual at the same time point. In the present study, we found a substantial reduction in the exposure of CoRbs of LT5.J4b12C Env in the presence of an excess molar concentration of sCD4, both when expressed as pseudotyped virus and as soluble trimeric protein, indicating that the overall conformation of LT5.J4b12C SOSIP.664 was not altered to the extent required for optimal exposure of CD4i epitopes that the mAbs can access despite the presence of residues required for CD4 engagement. EM analysis clearly demonstrated that LT5.J4b12C SOSIP.664 primarily preserves its prefusion conformation in the presence of excess CD4, indicating that it possesses a unique and intrinsic property in direct contrast to BG505 SOSIP.664 and B41 SOSIP.664 trimers examined under the same experimental condition. Interestingly, compared with BG505 and B41 Env trimers, LT5.J4b12C SOSIP.664 was found to dissociate faster post-CD4 engagement in BLI Octet analysis, which perhaps explains the basis of greater resistance of LT5.J4b12C Env trimers to undergo CD4-induced conformational changes. The observation that a vast majority (∼90%) of LT5.J4b12C SOSIP.664 trimers retain a prefusion conformation in the presence of sCD4 as determined by EM analysis but the pseudovirus shows productive infection in TZM-bl cells is intriguing. Unlike other enveloped viruses, HIV-1 Env spikes are present at very low density on virus particles, and it has been shown that very few functional spikes (≤4 spikes/virion) are sufficient for attachment and fusion (27–30). Therefore, we believe that a minor number of CD4-induced open Env trimers (∼10%) as determined by EM analysis are still sufficient to mediate productive infection of TZM-bl cells that express high levels of CD4 and CCR5 on their surface (31). Furthermore, the native Env spikes expressed on virus particles contain the cytoplasmic tail and membrane-proximal external region (MPER) (11), which are believed to influence the trimer structure associated with binding to CD4 and virus entry (32–35). Molecular modeling data indicate the presence of a unique C2V3 sequence in LT5.J4b12C Env that via conformational rearrangements mediates significant retraction of the C2 region along with displacement of the loop D of the viral Env essential for CD4 and VRC01 binding and that possibly structurally hinders the LT5.J4b12C Env and its weak interaction with CD4. In line with the above observation, LT5.J4b12C Env was indeed found to be inaccessible by the VRC01 mAb due to the presence of Glu^279^ residue in loop D. Susceptibility to VRC01, however, did not influence differential binding of LT5.J4b12C Env to CD4, suggesting that more than one CD4bs residue is associated with low affinity to CD4. The remarkable resistance of LT5.J4b12C SOSIP.664 to CD4-induced conformational changes coupled with our earlier observation (25) showing inability of LT5.J4b12C Env when expressed as pseudotyped virus infecting cells expressing very low molecules of CD4 on their surface indicates that CD4 having significantly lower expression on T cells *in vivo* (36) than that of established cell lines would possibly have minimal impact in altering LT5.J4b12C Env trimers in a physiologically relevant environment. Indeed, minimizing the degree of trimer conformational changes by selective modification has been demonstrated to decrease non-neutralizing antibody-directed responses (7) and hence been proposed to be a hallmark feature of experimental immunogens with reduced exposure of non-neutralizing epitopes. The ability of trimeric Env to undergo a very minimal or no CD4-induced conformational shift is desirable as immunogens to be tested in a non-human primate model and humans where gp120 binds CD4 with very high affinity (8). In summary, we report a HIV-1 B/C recombinant Env that in its soluble form retains the majority of its trimers in a native-like, prefusion conformation in the presence of CD4 and expresses epitopes that are selectively targeted by broadly neutralizing antibodies. Such features makes this trimeric Env an attractive component suitable for use as an antigen bait for bnAb isolation (37), further structural studies, and assessing its immunogenic potential in animal models.

## Experimental procedures

### Cells, DNA, plasma, proteins, and antibodies

293T and 293F cells were obtained from the American Type Culture Collection (ATCC), and TZM-bl cells were obtained from the National Institutes of Health AIDS Research Reagents and Reference Program. Plasmid DNA encoding BG505 SOSIP.664 with and without a C-terminal D7324 epitope tag was kindly provided by Prof John P. Moore, Weill Cornell Medical College, New York. The DNA plasmids encoding LT5.J4b and LT5.J12 *envs* (GenBank accession numbers FJ515875 and FJ515876, respectively) used to prepare chimeric LT5.J4b12C Env were described previously (25, 26). psG3Δenv was obtained from the National Institutes of Health AIDS Research Reagents and Reference Program. sCD4 (two and four domains; National Institutes of Health AIDS Research Reagents and Reference Program catalogue numbers 7356 and 4615, respectively) and mAbs used in this study were either obtained from the National Institutes of Health AIDS Research Reagents and Reference Program or obtained through the International AIDS Vaccine Initiative Neutralizing Antibody Center at the Scripps Research Institute, La Jolla, CA. Affinity-purified sheep anti-C5 antibody (targeting the D7324 epitope) was purchased from Aalto Bio Reagents, Inc.

### Codon optimization and preparation of LT5.J4b12C gp140 SOSIP.664 soluble protein

The LT5.J4b12C chimeric Env was prepared by swapping the C2V3 sequence of the LT5.J12 *env* into the LT5.J4b *env* (both of which were obtained from broadly neutralizing plasma of an antiretroviral therapy-naïve long-term non-progressor of Indian origin), which when expressed as pseudovirus showed susceptibility only to the neutralizing antibodies but not to the non-neutralizing antibodies and which was reported previously (25). The LT5.J4b12C SOSIP.664 gp140 construct was designed essentially as described by Sanders *et al.* (11). LT5.J4b12C *env* gene was codon-optimized by GeneArt (Thermo Fisher Inc.) and cloned into pcDNA3.1(+) using NheI and ApaI. The following modifications to the wild-type Env sequence were made: A501C, T605C, I559C (for trimer stabilization), and gp120–gp41 cleavage motif REKR changed to RRRRRR. The D7324 epitope sequence (GSAPTKAKRRVVQREKR) was added after residue 664 in gp41 ectodomain (ECTO) and preceding the stop codon to facilitate examining Env SOSIP binding by ELISA as described elsewhere (12). LT5.J4b12C SOSIP.664 was expressed by transient transfection of 293F cells, and the trimeric proteins were purified from culture supernatants using a PGT151 mAb affinity column. Bound proteins were eluted with 3 m MgCl_2_, dialyzed with phosphate-buffered saline (PBS; pH 7.4), and subsequently concentrated using Amicon ultracentrifuge filters (Millipore) with a 100-kDa cutoff to 0.5–1 ml. The purified proteins were further purified by size exclusion chromatography using a HiLoad Superdex 200 16/60 column (GE Healthcare). The purified proteins were snap frozen in liquid nitrogen and stored at −80 °C until further use. Purified SOSIP proteins were analyzed in a gradient 4–15% BN-PAGE (Mini-PROTEAN TGX^TM^, Bio-Rad). To analyze the degree of cleavage of SOSIP, the trimeric protein was incubated with 0.1 m dithiothreitol (DTT) before running SDS-PAGE under reducing conditions.

### D7324-capture ELISA

Binding of mAbs to the LT5.J4b12C SOSIP.664-D7324 trimeric protein was assessed essentially as described by Sanders *et al.* (11) in a sandwich ELISA. Briefly, high-binding microtiter plates (Nunc, Inc.) were first coated with D7324 antibody (Aalto Bio Reagents, Dublin, Ireland) at 10 μg/ml (100 μl/well) in coating buffer (150 mm Na_2_CO_3_, 350 mm NaHCO_3_, 30 mm NaN_3_, pH 9.6) at 4 °C overnight. Plates were washed thrice using PBS and subsequently blocked with 220 μl of 5% (w/v) nonfat milk in PBS and incubated at 37 °C for 1–1.5 h. After blocking and washing steps, purified LT5.J4b12C SOSIP.664-D7324 trimers were added at 500 ng/ml in PBS (100 μl/well) for 2–3 h. Unbound trimers were removed by washing three times with PBS, and PBS plus 3% (w/v) skimmed milk (250 μl/well) was added to further block nonspecific protein-binding sites. Different concentrations/dilutions of mAbs were added and incubated for 1.5–2 h at 37 °C. After three washes with PBS, 100 μl of anti-human HRP (Jackson ImmunoResearch Laboratories) diluted at 1:2000 were added and incubated at room temperature for 50 min to 1 h. The plates were then washed four times with PBS with Triton X-100 (0.05%, v/v). 100 μl of tetramethylbenzidine (TMB) substrate was added, and the reaction was stopped by adding 2 n H_2_SO_4_. Absorbance was measured at 450 nm.

### BN-PAGE gel shift assay

The Env–sCD4 interaction was assessed by BN-PAGE gel shift assay following essentially the method recently described by de Taeye *et al.* (7). Briefly, the purified LT5.J4b12C SOSIP.664 and BG505 SOSIP.664 trimeric proteins (2.5 μm each) were mixed with a 10-fold molar excess (25 μm) of two- or four-domain sCD4 for 1–1.5 h at 37 °C. The sCD4-treated proteins were mixed with loading dye, and samples were analyzed by BN-PAGE. The purified LT5.J4b12C and BG505 SOSIP.664 trimeric proteins without sCD4 pretreatment were used as controls.

### BLI

His_6_-tagged sCD4 (D1-D2) was immobilized on Ni-NTA sensors and dipped into varying concentrations (500, 250, 125, 62.5, 32.25, 16, and 8 nm) of either BG505 or LT5.J4b12C SOSIP.664 using an Octet Red96 instrument (ForteBio Inc.). In parallel, blank Ni-NTA sensors were also dipped into the same SOSIP samples to subtract background (nonspecific) binding of SOSIP Envs to the Ni-NTA biosensor. All samples were previously diluted in phosphate-buffered saline, pH 7.2, containing 0.01% (w/v) bovine serum albumin and 0.002% (v/v) Tween 20). Data were analyzed using the included Data Analysis software by subtracting reference curves and using a 1:1 binding model to fit the association and dissociation curves. A global fit was performed using all curves in which the concentration of SOSIP yielded a change in binding of at least 0.1 nm and a measurable dissociation.

### DSC

gp140 molecules were exchanged into phosphate-buffered saline, pH 7.2, using centrifugal filter units (Millipore) and diluted to 0.1–0.2 mg/ml. Samples were loaded onto a MicroCal VP-Capillary DSC instrument (Malvern) and subjected to a 20–90 °C ramp at 60°/h. Origin 7.0 software was used to subtract baseline measurements and to fit the melting curves using a non-two-state model. Reported *T_m_* values are for the tallest peak of each sample.

### Negative-stain EM

SOSIP trimers, either alone or preincubated with an ∼6-fold molar excess of two-domain sCD4 for 2 h (1.4 and 9 μm, respectively), were diluted to 0.01–0.03 mg/ml, applied to a carbon-coated Cu400 grid, and stained with 2% (w/v) uranyl formate as described previously (7). Data were collected on an FEI Tecnai Spirit T12 transmission electron microscope operating at 120 keV and equipped with a Tietz TVIPS complementary metal oxide semiconductor camera. A magnification of 52,000× was used, resulting in a physical pixel size at the specimen plane of 2.05 Å. Data processing and analysis methods have been reported elsewhere (7). Two-dimensional classifications were performed using RELION 2.0 (38).

### Site-directed mutagenesis

Point mutations were introduced in plasmid DNA constructs using the QuikChange II kit (Agilent Technologies Inc.) following the manufacturer's protocol, and introduction of specific substitutions was confirmed by sequencing as described previously (39).

### Preparation of Env-pseudotyped viruses

Env-pseudotyped viruses were prepared by the co-transfection of *env*-expressing plasmid with an *env*-deleted HIV-1 backbone plasmid (pSG3Δenv) into 293T cells as described before (39). Cell supernatants containing pseudotyped viruses were harvested 48 h post-transfection and subsequently stored at −80 °C until further use.

### Neutralization assay

Neutralization assays were carried out using TZM-bl cells as described before (40). Env-pseudotyped viruses were incubated with varying dilutions of antibodies and incubated for an hour at 37 °C in a CO_2_ incubator under humidified condition, and subsequently 1 × 10^4^ TZM-bl cells were added into the mixture in the presence of 25 μg/ml DEAE-dextran (Sigma). The plates were further incubated for 48 h, and the degree of virus neutralization was assessed by measuring relative luminescence units in a luminometer (Victor X2, PerkinElmer Life Sciences).

### Molecular modeling

Structural models of LT5.J4b, LT5.J12, and LT5.J4b12C Envs were generated through the SWISS-MODEL homology modeling server (41). Additional energy minimization was carried out using the CHARMM force field through Discovery Studio 3.5 (Accelrys Inc.). The quality of minimized structures was accessed by PROCHECK. Intersubunit contacts of HIV-1 YU2 Env-CD4 (Protein Data Bank code 4RQS) (42) were derived through the CONTACTS tool implemented in the CCP4 package (43). Structural superimposition, visualization, and creation of figures displaying the protein structures were done with PyMOL software (44). Multiple amino acid sequence alignments were made with the ClustalW tool using Mega (version 5.2) program software and analyzed in SeqPublish (www.hiv.lanl.gov). The VRC01 contact points were obtained from the CATNAP database (https://www.hiv.lanl.gov/content/immunology/neutralizing_ab_resources.html).

## Author contributions

J. B. and R. K. conceived the study and designed the experiments. R. K. and V. K. carried out protein purification, expression, and characterization work. S. P., S. D., and R. K. carried out virus work and sequence analysis. G. O. and A. B. W. designed EM experiments. G. O. and L. G. H. carried out EM and BLI Octet binding experiments. G. O. and A. B. W. analyzed EM and Octet binding data. T. S. carried out the structure-based bioinformatics analysis and molecular modeling. J. B. and R. K. wrote the manuscript with the help of all the co-authors.

## Footnotes

2 The abbreviations used are:

HIV-1: human immunodeficiency virus, type 1
Env: HIV-1 envelope
bnAb: broadly neutralizing antibody
sCD4: soluble CD4
CoRbs: co-receptor (CXCR4/CCR5)-binding site
CD4bs: CD4-binding site
CD4i: CD4-induced
DSC: differential scanning calorimetry
Ni-NTA: nickel-nitrilotriacetic acid
BLI: biolayer interferometry
BN: blue native.
